# Creatine and Taurine as Novel Competitive Inhibitors of Acetylcholinesterase: A Biochemical Basis for Nutritional Modulation of Brain Function

**DOI:** 10.3390/ijms262311309

**Published:** 2025-11-22

**Authors:** Paweł Adamski, Łukasz Szeleszczuk, Marcin Gackowski, Błażej Grodner

**Affiliations:** 1 Department of Biochemistry and Pharmacogenomics, Medical University of Warsaw, 1 Banacha Str., 02-097 Warsaw, Poland; s088815@student.wum.edu.pl; 2 Department of Organic and Physical Chemistry, Medical University of Warsaw, 1 Banacha Str., 02-097 Warsaw, Poland; lukasz.szeleszczuk@wum.edu.pl; 3 Department of Toxicology and Bromatology, Faculty of Pharmacy, L. Rydygier Collegium Medicum in Bydgoszcz, Nicolaus Copernicus University in Torun, 2 Jurasza Str., 85-089 Bydgoszcz, Poland; marcin.gackowski@cm.umk.pl

**Keywords:** acetylcholinesterase, creatine, taurine, enzyme inhibition, competitive inhibitor, neuroprotection

## Abstract

Acetylcholinesterase (AChE) is a key enzyme responsible for terminating cholinergic neurotransmission by hydrolyzing acetylcholine. While clinically approved AChE inhibitors such as donepezil, rivastigmine, and galantamine are used in the symptomatic treatment of Alzheimer’s disease and related dementias, little is known about the modulatory effects of common dietary compounds on AChE activity. In this study, we investigated the influence of creatine (CR) and taurine (TA)—two widely consumed nutritional supplements with reported neuroprotective and cognitive-enhancing properties—on AChE. Enzyme kinetics were evaluated using a modified Ellman’s method, and Lineweaver–Burk analyses revealed that both CR and TA act as competitive inhibitors. Calculated parameters (Km, Vmax), inhibition constants (Ki), and half maximal inhibitory concentrations (IC_50_) consistently indicated stronger potency for CR (IC_50_ = 0.0056 ± 0.00018 mM) compared to TA (IC50 = 0.0097 ± 0.00035 mM). To complement the experimental data, molecular docking was performed using two crystal structures of human AChE. Docking confirmed that both ligands preferentially occupy the active-site region in a manner consistent with competitive inhibition, with CR showing more favorable binding scores than TA. Although markedly weaker than clinical drugs, these findings provide the first biochemical and in silico evidence that CR and TA directly interact with AChE, suggesting subtle cholinergic modulation relevant to cognitive function and neuroprotection.

## 1. Introduction

Acetylcholinesterase (AChE; EC 3.1.1.7) is a hydrolytic enzyme of the cholinesterase family that plays a crucial role in cholinergic neurotransmission. It catalyzes the hydrolysis of acetylcholine (ACh) and other choline esters, producing choline and acetic acid. AChE is primarily located in the postsynaptic membranes of cholinergic synapses in both the central and peripheral nervous systems, where it facilitates the rapid termination of neural signaling [1,2]. Pharmacologically, inhibition of AChE increases synaptic ACh levels, thereby enhancing neurotransmission. Acetylcholinesterase inhibitors (AChEIs) such as donepezil, galantamine, and rivastigmine are widely used in the treatment of Alzheimer’s disease and other dementias, improving cognitive performance and alleviating neuropsychiatric symptoms [3,4,5,6,7].

**Figure 1:**
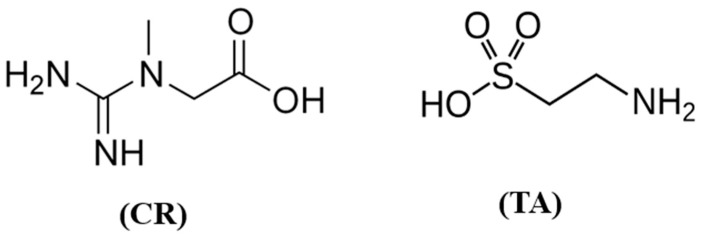
Chemical structures of Creatine (CR) and Taurine (TA).

Creatine (N-amidinoacetate) (CR) (Figure 1) is an endogenous guanidino compound synthesized in the liver, kidneys, and pancreas, and transported to high-energy-demand tissues, including the brain. Its primary function is to maintain cellular energy homeostasis via the phosphocreatine buffer system [8,9]. Evidence suggests that creatine may influence the cholinergic system, potentially through improved adenosine triphosphate (ATP) availability in neurons and indirect modulation of ACh synthesis and release [10,11]. Deficiency of brain creatine is associated with severe developmental and psychiatric disorders, many of which can be improved by supplementation [12]. Moreover, creatine has been linked to enhanced cognitive performance under metabolic stress, sleep deprivation, or hypoxia [13,14]. Supplementation has been shown to improve reaction time, memory, and learning capacity, particularly in older individuals [15,16,17,18]. Some studies propose a direct role of creatine as a neurotransmitter within the central nervous system (CNS) [19].

Taurine (2-aminoethanesulfonate) (Figure 1) is a non-protein sulfur-containing amino acid abundant in the brain, retina, and heart. It exerts multiple physiological effects, including osmoregulation, membrane stabilization, modulation of ionic conductance, and antioxidant protection [20,21]. In the CNS, taurine functions as a neuromodulator, promoting neuronal hyperpolarization, likely through activation of chloride channels in the cerebellum and hippocampus [22,23]. Its neuroprotective potential has been associated with the attenuation of β-amyloid toxicity, regulation of intracellular calcium, and reduction in oxidative stress [24,25].

Despite their broad physiological relevance, there is currently no direct experimental evidence for the effect of creatine and taurine—either endogenous or supplemented—on AChE activity in vitro or in vivo. Existing studies mainly address their roles in neuroprotection, energy metabolism, and general CNS function, with only limited indications of possible indirect effects on cholinergic signaling [10,11].

Given their widespread and often combined use in dietary supplements, along with the growing evidence of their neuromodulatory functions, it is essential to examine their potential influence on AChE activity. The present study was therefore designed to investigate the inhibitory effects of creatine and taurine on AChE, combining enzymatic assays with molecular docking to provide complementary biochemical and computational insights.

## 2. Results

To assess whether CR and TA act as inhibitors of AChE, their effects on enzymatic activity were examined.

### 2.1. Experiments

The experiments were performed according to the protocol described in our previous study [26]. The reaction system contained acetylthiocholine (ACth) as the substrate, 5,5′-dithio-bis-2-nitrobenzoic acid (DTNB) as the chromogenic reagent, and purified AChE in the absence or presence of the tested compounds (CR, TA). The reaction product, 5-thio-2-nitrobenzoic acid (TNB), was quantified using a modified Ellman’s method. Enzyme activity was first evaluated over a range of substrate concentrations (0.39–200 mM ACth) to establish baseline kinetics. Subsequently, inhibition studies were performed at two fixed concentrations of CR or TA (0.15 and 1.5 mM) while varying substrate concentration, enabling the determination of kinetic parameters and inhibition constants.

### 2.2. Analysis

**Figure 2:**
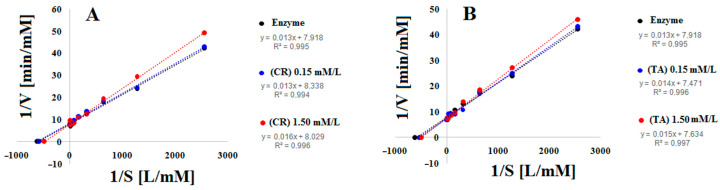
Lineweaver–Burk plots for AChE systems with inhibitors (CR) (**A**) and (TA) (**B**) at 0.15 mM and 1.5 mM.

In the first stage, kinetic studies were performed to characterize the mechanism of AChE inhibition by CR and TA. Lineweaver–Burk plots were generated from steady-state inhibition data (Figure 2), showing the relationship between the reciprocal of reaction rate and substrate concentration. In the absence of inhibitors, the correlation coefficient for AChE was 0.9952 with a slope of 0.0135. At 1.5 mM, CR and TA maintained similarly high correlation coefficients (0.9967 and 0.9970, respectively) but displayed steeper slopes (0.0163 and 0.0152), indicating reduced substrate affinity in the presence of either compound (Table 1, Figure 2).

The Lineweaver–Burk plots for CR and TA intersected at a common point on the y-axis, confirming that both compounds act as reversible competitive inhibitors of AChE. This conclusion was further supported by kinetic parameters: the K_m_ increased, while the V_max_ remained unchanged in the presence of the inhibitors. Creatine emerged as the more potent inhibitor, displaying a slightly higher K_m_ (2.03 × 10^−3^ mM) and a steeper slope (1.55 × 10^−3^ min) compared with taurine (2.00 × 10^−3^ mM; 1.53 × 10^−3^ min) (Table 1, Figure 2).

**Figure 3:**
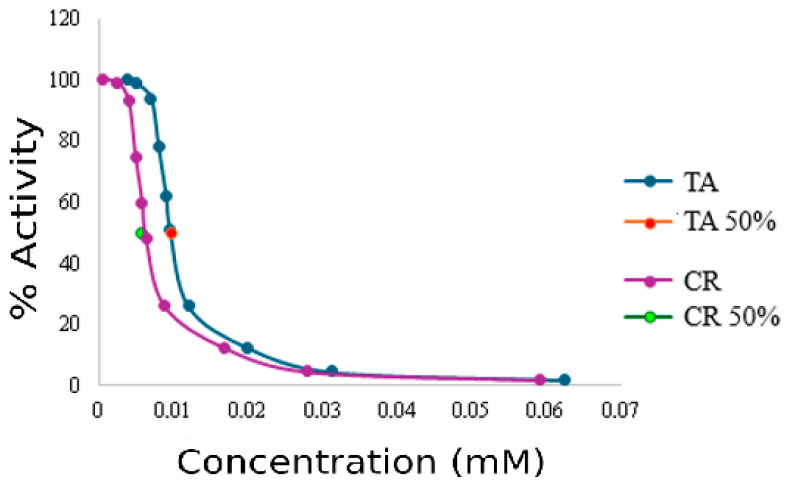
Graphical IC_50_ determination for inhibitors CR and TA.

To further quantify their inhibitory potency and binding affinity, Michaelis–Menten constants (Km), maximum velocities (V_max_), inhibition constants (K_i_), and half-maximal inhibitory concentrations (IC_50_) were determined at two inhibitor concentrations for both CR and TA (Table 1, Figure 3).

The observed increases in K_m_ values for creatine (CR; 1.90–2.03 × 10^−3^ mM) and taurine (TA; 1.80–2.00 × 10^−3^ mM), together with nearly constant V_max_ values (0.131–0.133 mM/min), confirmed that both compounds act as competitive inhibitors of AChE (Table 1). Among them, CR produced the greater shift in Km, consistent with stronger competition for the active site.

Lineweaver–Burk slopes (K_m_/V_max,_ min) also increased with inhibitor concentration, reaching 1.43 × 10^−3^–1.55 × 10^−3^ min for CR and 1.36 × 10^−3^–1.53 × 10^−3^ min for TA (Table 1, Figure 2). This pattern further supports the conclusion that CR is the more potent inhibitor.

Binding strength was assessed through inhibition constants (K_i_), calculated at 0.15 and 1.5 mM inhibitor concentrations. CR showed markedly lower K_i_ values (199.3) compared with TA (273.9), indicating tighter binding to the AChE active site (Table 1).

The half-maximal inhibitory concentration (IC_50_) provided additional confirmation of potency. CR inhibited AChE at 0.0056 ± 0.00018 mM, whereas TA required a higher concentration (0.0097 ± 0.00035 mM) (Table 1, Figure 3).

In summary, both CR and TA act as competitive inhibitors of AChE, with creatine consistently showing greater affinity and inhibitory strength than taurine. Notably, the kinetic parameters are in excellent agreement with molecular docking simulations, which likewise predicted stronger binding of CR.

### 2.3. Discussion

Analysis of the kinetic parameters confirmed the competitive nature of inhibition. In the presence of CR and TA, K_m_ values increased (CR: 1.90–2.03 × 10^−3^ mM; TA: 1.80–2.00 × 10^−3^ mM), while V_max_ remained unchanged (~0.131–0.133 mM/min). This pattern is consistent with competitive inhibition, where the inhibitor competes with the substrate for the enzyme’s active site. The larger K_m_ shift observed for CR suggests stronger interference with substrate binding.

Lineweaver–Burk slopes (Km/Vmax, min) likewise supported this conclusion, being higher for CR (1.43 × 10^−3^–1.55 × 10^−3^ min) than for TA (1.36 × 10^−3^–1.53 × 10^−3^ min) as inhibitor concentration increased. These results align with K_i_ and IC_50_ values, which likewise indicated greater potency of CR (K_i_: 199; IC_50_: 0.0056 mM) compared with TA (K_i_: 274; IC_50_: 0.0097 mM).

When compared to clinically established AChE inhibitors, both compounds are clearly much weaker. Donepezil (K_i_ = 0.070; IC_50_ ≈ 0.027 μM) is over 270,000-fold more potent than CR and 359,000-fold more potent than TA. Physostigmine (K_i_ = 0.030; IC_50_ ≈ 0.18 μM) is ~31,000-fold and ~54,000-fold stronger, respectively. In contrast, rivastigmine (K_i_ = 15.0; IC_50_ ≈ 71 μM) is less active, making CR and TA 79- and 137-fold more effective than this drug. Compared with selected donepezil derivatives, CR and TA remain 5–10 times less potent.

A comparison of binding affinities further confirmed this trend. The K_i_ values of CR and TA were several orders of magnitude weaker than those reported for donepezil, physostigmine, and rivastigmine across different brain regions.

Taken together, the data indicate that while CR and TA are far less potent than leading therapeutic inhibitors, creatine consistently shows stronger binding and inhibitory activity than taurine.

It should be clearly noted that both CR and TA have significantly lower AChE inhibitory potential compared to previously used inhibitors, such as donepezil or physostigmine [27,28,29,30]. However, taking both of these substances in commonly used stimulants, in amounts recommended by the manufacturers, demonstrates a low but measurable inhibitory effect of both CR and TA on AChE activity.

Although the present study demonstrates that creatine and taurine can directly inhibit AChE activity in vitro, the biological relevance of such inhibition is likely limited under physiological conditions. The inhibitor concentrations observed in vitro exceed the plausible free concentrations of creatine and taurine that are achievable in vivo, which are tightly regulated. Moreover, both compounds are zwitterionic and display poor affinity for the hydrophobic gorge of AChE, suggesting that their interactions are transient and of modest energetic stability. Consequently, it is unlikely that creatine or taurine significantly modulates cholinergic neurotransmission in vivo through direct enzyme inhibition. Instead, their well-documented neuroprotective and cognitive effects probably arise from indirect mechanisms, such as improved cellular energetics (for creatine) or regulation of calcium homeostasis and oxidative balance (for taurine). Explicitly acknowledging this limitation strengthens the interpretation that the observed inhibitory activity represents a biochemical curiosity rather than a physiologically significant effect, while still providing helpful insight into potential molecular interactions of these widely consumed nutritional supplements.

### 2.4. Molecular Docking

**Figure 4:**
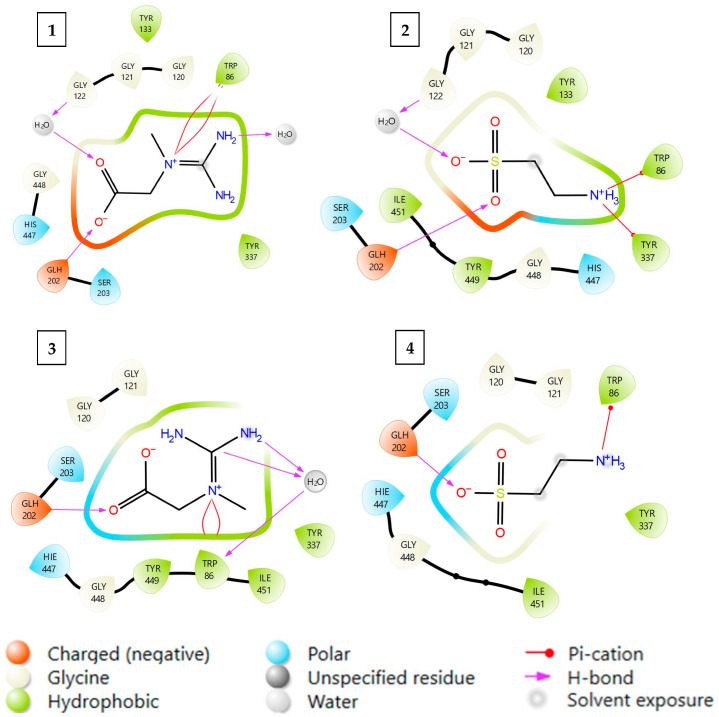
Binding modes of CR and TA with AChE obtained from molecular docking. Description of the models (**1**–**4**) is presented in Table 2.

In this work, molecular docking was employed to provide insight into the interactions of CR and TA with AChE. Two crystal structures of the human enzyme (PDB IDs: 4EY6 and 7XN1), co-crystallized with competitive (galantamine) and non-competitive (tacrine) inhibitors, respectively, were used as docking targets. The application of two distinct receptor conformations enabled us to evaluate whether the studied ligands favor binding patterns consistent with competitive or non-competitive inhibition. This approach had been successfully applied in our recent study on thiamine (T) and thiamine pyrophosphate (TPP), where it allowed us to characterize their inhibitory mode [26]. Here, the obtained docking results for CR and TA (Table 2, Figure 4) serve as complementary in silico validation of their experimentally observed competitive inhibition of AChE.

Docking simulations of the tested ligands into both AChE crystal structures revealed notable differences depending on the reference complex. When docked to the enzyme structure co-crystallized with the competitive inhibitor galantamine, the ligands produced markedly higher docking scores and more negative MM/GBSA ΔG values, with CR achieving a higher score than TA. In contrast, docking to the tacrine-bound structure, representing a non-competitive inhibitor complex, yielded substantially lower scores and less negative MM/GBSA ΔG values, indicating that this binding mode is less favorable in terms of protein–ligand interactions. The docking scores and MM/GBSA ΔG values obtained for CR were more negative than those for TA, indicating stronger binding in the case of CR, which is in agreement with the experimental results.

## 3. Materials and Methods

### 3.1. Reagents and Chemicals

Human acetylcholinesterase (AChE; EC 3.1.1.7), acetylthiocholine iodide (ACthI), 5,5′-dithio-bis-2-nitrobenzoic acid (DTNB), 5-thio-2-nitrobenzoic acid (TNB), (CR), and (TA) were purchased from Sigma-Aldrich (Poznań, Poland). All reagents were of analytical grade, with a purity of greater than 98%.

### 3.2. Instrumentation

Kinetic measurements were performed using a Cole-Parmer SP-200-UV benchtop UV/Vis spectrophotometer (Cole-Parmer Instrument Company, LLC, Vernon Hills, IL, USA) (range: 198–1000 nm).

### 3.3. Preparation of Standards

Stock solutions of CR and TA (2 M) were prepared in deionized water. A 200 mM ACthI stock solution was obtained by dissolving 2.8918 g of ACthI in 50 mL of phosphate buffer (100 mM, pH 7.5). Serial dilutions of ACthI were used to prepare working solutions ranging from 0.39 to 200 mM.

### 3.4. Sample Preparation

AChE was dissolved in phosphate buffer (100 mM, pH 7.5), aliquoted, and stored at −20 °C. Before each experiment, aliquots were thawed, and enzyme activity was verified. Enzyme activity was measured using the Ellman method [30], with modifications by Kaizer et al. [31]. Reaction mixtures contained phosphate buffer, ACthI, inhibitor solution (CR or TA), DTNB, and water, and were incubated at 25 °C before the reaction was initiated by adding AChE. AChE was added at a final activity of 0.025 U/mL (50 µL of a 1 U/mL stock into a 2.0 mL reaction volume). Product formation was monitored spectrophotometrically at 412 nm.

### 3.5. Molecular Modelling

#### 3.5.1. Ligand Preparation

The 3D structures of creatine (Refcode: JOHJIB05) and taurine (Refcode: TAURIN15) were retrieved from the CCDC database. Before docking, their geometries were processed using the LigPrep tool (Schrödinger Suite, New York, NY, USA, Release 2021-1), with the OPLS4 force field applied. Possible protonation and tautomeric states at physiological pH (7.5 ± 1.0) were generated using the Epik module, ensuring that zwitterionic forms relevant under physiological conditions were included in the docking workflow.

#### 3.5.2. Protein Preparation

The crystal structures of human AChE bound with galantamine (PDB ID: 4EY6) and tacrine (PDB ID: 7XN1) were obtained from the Protein Data Bank. Structures were preprocessed using the Protein Preparation Wizard, which included the addition of hydrogen atoms and optimization of hydrogen-bonding networks. Protonation states of ionizable residues were adjusted using the PROPKA algorithm at pH 7.5. Energy minimization was performed using the OPLS4 force field until the RMSD of heavy atoms converged to 0.3 Å. The prepared models were then used to generate docking grids.

#### 3.5.3. Docking Protocol

For each AChE structure, docking grids were defined based on the centroid of the co-crystallized inhibitor, with inner and outer cubic boxes of 10 × 10 × 10 Å and 20 × 20 × 20 Å, respectively. Docking of CR and TA was performed using the Glide XP (extra precision) protocol, which employs flexible ligand sampling and the OPLS4 scoring function. Epik penalties for ionization states were excluded from the final scoring.

#### 3.5.4. MM/GBSA Calculations

To evaluate ligands’ affinity toward the enzyme in silico, we determined the binding free energies (ΔG_bind_). These calculations were performed using the MM/GBSA approach, implemented in the Prime module, which provides refined post-docking estimates of interaction strength. The method employed the OPLS-2005 force field, along with the VSGB implicit solvent model, to assess the energetic changes accompanying protein–ligand association.

Binding free energies were obtained according to:

$$\Delta G_{\mathrm{bind}}=G_{\mathrm{complex}}^{\mathrm{opt}}-(G_{\mathrm{protein}}^{\mathrm{opt}}+G_{\mathrm{ligand}}^{\mathrm{opt}})$$

For each individual state (complex, protein, and ligand), the total free energy was determined as the sum of molecular mechanics contributions, solvation terms, and entropic factors:

$$G=G_{\mathrm{int}}+G_{\mathrm{Coulomb}}+G_{\mathrm{vdW}}+G_{\mathrm{GB}}+G_{\mathrm{lipo}}-TS$$

Here, *T* denotes temperature and *S* is the configurational entropy. The molecular mechanics terms include covalent bonding (bonds, angles, dihedrals), electrostatic interactions, and van der Waals forces. The solvation component consists of a polar contribution (G_GB_), estimated using the generalized Born model, and a non-polar (G_lipo_) term derived from the solvent-accessible surface area (SASA).

### 3.6. Kinetic and Statistical Analysis

Two inhibitor levels (0.15 and 1.5 mM) were preselected based on preliminary concentration–response screens to bracket the inhibitory range and provide well-separated slopes for competitive inhibition fitting, while maintaining assay linearity and avoiding matrix effects at higher solute levels. Initial reaction rates (v, mM/min) were derived from the initial slope of A412 time traces (absorbance at 412 nm) recorded with the modified Ellman assay at 25 °C, converting ΔA412/Δt to concentration change using the molar absorptivity of TNB at 412 nm and the effective optical pathlength. Lineweaver–Burk plots (1/v vs. 1/[S]) were fitted by linear regression; the slope and intercept were used to determine K_m_ and V_max_. K_i_ values were derived from Lineweaver–Burk secondary plots under a competitive-inhibition model. IC_50_ values were obtained from concentration–response curves by nonlinear regression (four-parameter logistic model, 95% CI). Data are reported as mean ± SD from *n* = 6 independent replicates. Curve fits and parameter estimates (IC_50_, K_m_, V_max_, K_i_) were obtained by regression as specified above; goodness-of-fit is reported as R^2^. Where applicable, curve/parameter comparisons used the extra sum-of-squares F-test (two-sided, α = 0.05). Analyses were performed in GraphPad Prism v10.6.1. (GraphPad Software, San Diego, CA, USA).

## 4. Future Directions and Limitations of the Current Study

Emerging evidence indicates that several nutritional compounds exhibit hormetic properties, characterized by beneficial cellular stimulation at low or moderate doses and inhibition or toxicity at higher doses. Creatine and taurine have been described as “hormetic nutrients” capable of modulating antioxidant and anti-inflammatory pathways in a dose-dependent manner, supporting adaptive cellular responses that may contribute to neuroprotection. Recent studies demonstrate that moderate concentrations of such nutrients can activate endogenous redox-regulating and anti-inflammatory cascades across diverse experimental models [32,33]. In this context, the inhibitory effects on AChE observed at 0.15–1.5 mM may reflect a broader dose-responsive pattern consistent with hormetic modulation of neuronal function.

Although our results demonstrate clear in vitro and in silico evidence of competitive AChE inhibition by creatine and taurine, several limitations should be considered. First, the inhibitor concentrations required to observe measurable effects exceed the free physiological concentrations achievable in vivo, where both compounds are tightly regulated. Second, the zwitterionic and highly polar nature of CR and TA likely limits their affinity for the hydrophobic catalytic gorge of AChE under physiological conditions. Third, the study did not include cell-based or in vivo models, which are necessary to establish whether the observed inhibitory effects have functional relevance for cholinergic neurotransmission. Therefore, the biological significance of direct AChE inhibition by CR and TA remains uncertain and may be overshadowed by their well-documented indirect neuroprotective mechanisms.

## 5. Conclusions

This study examined the inhibitory effects of CR and TA, compounds not previously described as inhibitors, on AChE. Both compounds acted as competitive inhibitors, with CR exhibiting greater potency than TA. The results showed that the nature and structure of the molecule influenced the inhibitory potency. Importantly, molecular docking provided complementary support for the experimental findings, demonstrating that CR and TA bind within the active-site region of AChE, with CR displaying stronger predicted binding interactions. Although both substances are significantly weaker inhibitors compared to clinically established drugs, the combined kinetic and docking data consistently indicate that creatine is a more effective competitive inhibitor than taurine. Given that both compounds are consumed in relatively large quantities as dietary supplements, they may exert some inhibitory effect on acetylcholinesterase activity.

Although the present study provides clear in vitro and in silico evidence of competitive AChE inhibition by creatine and taurine, further research is warranted to determine whether these effects are relevant under physiological conditions. Future studies should therefore incorporate cell-based assays and in vivo models to assess the potential impact of dietary or pharmacological concentrations of these compounds on cholinergic neurotransmission and cognitive function.

## Figures and Tables

**Table 1:** The analytical data describing the effects of acetylcholinesterase inhibition by CR and TA.

	Control (Enzyme Only)	Creatine 0.15 mM	Creatine 1.5 mM	Taurine 0.15 mM	Taurine 1.5 mM
Slope, K_m_/V_max_ [min]	1.32 × 10^−3^ ± 0.002	1.43 × 10^−3^ ± 0.003	1.55 × 10^−3^ ± 0.005	1.36 × 10^−3^ ± 0.001	1.53 × 10^−3^ ± 0.004
K_m_ [mM]	1.73 × 10^−3^ ± 1.47 × 10^−5^	1.90 × 10^−3^ ± 1.52 × 10^−5^	2.03 × 10^−3^ ± 1.37 × 10^−5^	1.80 × 10^−3^ ± 1.43 × 10^−5^	2.00 × 10^−3^ ± 1.41 × 10^−5^
V_max_ [mM/min]	0.131 ± 0.0014	0.133 ± 0.0015	0.131 ± 0.0014	0.132 ± 0.0015	0.131 ± 0.0013
K_i_ [µM]		199.348 ± 5.21		273.905 ± 5.94	
IC_50_ [µM]		0.0056 ± 0.00018		0.0097 ± 0.00035	

Values are mean ± SD (*n* = 6).

**Table 2:** Molecular docking results for the complexes formed between CR, TA, and human AChE.

Protein PDB Code	Cocrystalised Inhibitor	Docked Ligand	Model	GLIDE XP Score	MM-GBSA [Kcal/Mol]
4EY6	Competitive Galantamine	Creatine CR	1	−7.315	−34.582
		Taurine TA	2	−6.501	−28.971
7XN1	Non-competitive Tacrine	Creatine CR	3	−3.247	−24.536
		Taurine TA	4	−2.034	−19.808

## Data Availability

The original contributions presented in this study are included in the article. Further inquiries can be directed to the corresponding author.
